# Evaluation of the impact of a chronic disease scheme reimbursing medical costs of patients with diabetes in Anhui province, China: a follow-up study

**DOI:** 10.1186/s12889-016-3643-3

**Published:** 2016-09-15

**Authors:** Qicheng Jiang, Zhen Jiang, Zhang Xin, Nicola Cherry

**Affiliations:** 1 School of Public Health, Anhui Medical University, Anhui, China; 2 School of Health Management, Anhui Medical University, Anhui, China; 3 National HIV/AIDS Center, Anhui, China; 4 Anhui Provincial Hospital, Anhui, China; 5 Division of Preventive Medicine, University of Alberta, 5-22 University Terrace, 8303-112 St, Edmonton, AB T6G 2 T4 Canada

**Keywords:** China, NCMS, Chronic disease scheme, Diabetes, Quality of life

## Abstract

**Background:**

Although many studies have investigated the relationship between the introduction of the New Cooperative Medical Scheme (NCMS) in rural China in 2003 and increased use of medical services, the effect on health status, objectively measured, is seldom reported. In Anhui Province a chronic disease scheme (CDS) for reimbursing part of the cost of outpatient care is designed to improve management of those with chronic conditions, including diabetes.

**Methods:**

A follow-up study was designed in which patients with diabetes aged 40–70 years who had recently (in 2010) been granted a chronic disease card were individually matched on age, sex and village with a patient with diabetes not yet in the scheme. Each subject gave a fingertip sample of blood to give the concentration of glycosylated hemoglobin (HbA1c), a measure indicating blood glucose control during the previous 3 months. This measure was made on recruitment and at 12 month follow-up: information on use of health services, quality of life and financial burden was also collected at the two contacts.

**Results:**

Of 602 pairs initially recruited, 528 pairs were contacted at follow-up and are the subject of this report. To distinguish between outcomes associated with application and those of membership of the scheme, the primary analysis was of 256 pairs in which one had been a member of the CDS throughout and the other never applied. No difference between pairs on HbA1c was found either at recruitment or follow-up but those in the CDS reported more hospital visits, more tests and more use of high level hospitals. However they had poorer scores on quality of life scales (SF-12, EQ-5D) and were more likely to report that the financial costs were very burdensome. Those recently applying for the scheme, or being accepted since recruitment, had lower HbA1c scores.

**Conclusions:**

On-going membership of the CDS was associated with increased use of services but this did not appear to result in better management of blood glucose or improved quality of life. Those who had recently joined the scheme had signs of improvement, suggesting a need for active follow-up to maintain and reinforce early gains.

## Background

The new cooperative medical scheme (NCMS) for rural China was launched in 2003 to protect against impoverishment arising from catastrophic health expenditures and to improve the health status of rural residents. By 2012 almost all rural residents chose to join the scheme [1]. There have been a number of studies of the impact of the scheme on access to care, on patterns of care (including medications) and on the effectiveness in reducing the financial burden of catastrophic illness [2–5]. The impact on the health of participants has been much less well documented, in part because of difficulties in measuring effects of health care objectively. A recent systematic review found 12 studies of the relationship between NCMS membership and health outcome: all used reports of sickness or injury in the last 2–4 weeks or self-reports of health status. Conclusions were inconsistent, with no clear evidence that the NCMS improves health outcomes [6]. A clinical outcome, the control of hypertension, was used in one study to assess the effectiveness of the NCMS but concluded that NCMS membership contributed little [7]. There have been no studies using the success in controlling diabetes as a measure of the effectiveness of the NCMS: an earlier report considered the relation between reimbursement schemes and use of health services using diabetes as a ‘tracer’ condition but did not assess health outcome. [8] Diabetes, however, is particularly well suited for the assessment of clinical impact. It is common, has serious outcomes and has a biological marker that reflects the success of interventions. HbA1c measured from a blood sample, reflects the closeness of control of blood glucose in the preceding months. This provides an opportunity to evaluate the effect of health insurance during a relatively short period of follow-up. The present report considers the impact on diabetic control of a chronic disease program put in place in Anhui province as one part of that province’s NCMS.

The national model for the NCMS has common features across the country, including compensation for hospital inpatient stays and of outpatient costs for those with chronic disease. Each province has freedom to institute its own programs within these general guidelines. In Anhui province a chronic disease scheme (CDS) was added to the basic provisions of the NCMS. The study reported here was begun in 2010 and assessed the impact of membership of the CDS on patients diagnosed with diabetes. The scheme in place at that time paid 40 % of the costs of outpatient care and medications, to a maximum of 2000–3000 yuan/year (depending on county). There was, at the time, no clear clinical guideline as to treatment or frequency of follow-up.

In order to be accepted for the CDS a patient with diabetes had to be a member of the NCMS (a rural resident of Anhui not eligible for other health insurance schemes) whose diagnosis of diabetes had been confirmed by a specialist physician and who had been prescribed medication for this condition. The study reported here aimed to determine whether, having allowed for any important between group differences, those in the CDS had better control of their diabetes, more extensive diabetes related care, or better quality of life than those who had not yet joined the scheme.

## Methods

### The study setting

Anhui is an in-land province in Eastern China; Hefei, the capital, lies 550 miles south of Beijing. Until recently Anhui has been one of China’s poorer areas. Although Hefei is now booming, there are still many poor counties with rural inhabitants very largely working in agriculture and with low household incomes. The north of the province is flat and densely populated, with the south and west more mountainous and with difficult access. In 2010, when this study was begun, the overall population was close to 60 million. The province had 105 county-level divisions covered by 108 NCMS offices.

### Study design

This was a longitudinal (follow-up) study of two cohorts of patients diagnosed with diabetes living in six counties of Anhui. Within each county, patients with diabetes aged 40–70 years with chronic disease cards (‘cases’) were individually matched with patients diagnosed with diabetes without cards (‘referents’), on age, sex and village. Information was collected at baseline (close to the date at which the card of the case was issued) and after 12 months. Cross-over was allowed: someone not in the scheme could join, or someone in the scheme could leave without being excluded from the study. The study was designed to be analysed using a case-referent approach.

### Choice of study areas

In order to identify counties eligible for inclusion, the provincial health bureau approached 87 NCMS offices asking for numbers of patients in the chronic disease scheme and the proportion of patients for which age was recorded: 67 responded. Of these, 17 had age data for at least 90 % of patients for whom a chronic disease card had been approved. Counties were then eliminated if road access was very difficult or if, from past experience, collaboration was very unlikely. Six counties were chosen from the remaining pool, two with high rates of issuing cards, two with moderate rates and two with low. As no county from the north of the province was chosen by this approach, one county with age present for >80 % of those in the CDS and a moderate rate of issuing cards was substituted.

For the six counties identified in this way, further information was sought on the number of chronic disease registrations for diabetes and the date at which the scheme for diabetes was implemented. Resources allowed recruitment of 600 cases (card registrations) overall, and a target for each county was set, reflecting the number of new registrations/month. Information on the six counties and their participation in the CDS scheme is given in Table 1. To obtain a CDS card, the patient was assessed by a diabetic specialist and paid out-of-pocket for this at, depending on county, a county-level or township hospital, but not at a village clinic. In some counties applications for a CDS card could be made only once a year, in others at any time, with similar variations in frequency of reimbursement. The year in which the CDS scheme was first implemented ranged from 2006–2009, but no county had completely identified all patients with diabetes potentially eligible for the scheme by the time of the study.

**Table 1:** Counties included in the study

County	Population in NCMS	Year in which started CDS	Number of patients with diabetes in CDS to 2009	Rate of recruitment to CDS	Timing of application to CDS	Level of hospital accepting application	Timing of reimbursement	Reimbursement ceiling
A	221,955	2006	360	Mod	Anytime	County	2× year	2500
B	592,122	2006	522	Low	1× year	Township	Anytime	2000
C	319,328	2007	610	Mod	Anytime	County	1× year	3000
D	238,096	2009	514	High	4× year	Township	Anytime	2500
E	1,289,211	2008	800	Low	1× year	Township	2× year	2000
F	772,116	2009	2008	High	1× year	Township	1× year	2500

### Identification of patients with and without a chronic disease registration

In each of the 6 counties the NCMS staff listed all patients with diabetes in the CDS. The name, age, sex and phone number of those first receiving a card in 2010 were extracted, together with, in 4 counties, the most recent registrations from 2009. It was necessary to involve the township health centre (THC) for each village containing a case: for efficiency all potential cases (patients with CDS cards) within a single village were identified and the village clinic doctor acting as administrator (VCDA) was contacted by the THC. The VCDA then provided the ages, where missing, of potential cases. Because of serious logistic difficulties in carrying out the fieldwork, townships close to the county town were approached first, with more distant areas being included only if the target number of cases for the county could not be met in the more proximal villages.

There was no central register of patients with diabetes not registered for the CDS. In each village with an eligible case the VCDA listed all patients with diabetes aged 40–70 years known to the clinic. Those with a CDS card were then identified by the team from the lists supplied by the NCMS office and the remaining, potential referents, matched within village by age and sex to the identified cases. Where more than one referent (a patient with diabetes not in the CDS) was available, the one closest in age was approached first.

### Recruitment

Where possible patients both with and without a CDS card met with the research team at the village clinic and recruitment procedures and baseline data collection carried out there. Where a potential subject did not want to go to the clinic, the clinic VCDA went with the research team to the subject’s home. As the team could only be in the village for, at most, a few days, if a case could not be contacted in that time he or she was dropped from the study and replaced by another patient with a CDS card. If a referent could not be contacted and a second matched referent was available, this one was used. Only pairs (case and referent) were retained for the study.

Following an explanation of the project by the research staff the subject was asked to read an information sheet and sign consent. For illiterate subjects the information sheet and consent form were read to them and consent signified by a thumb print.

### Contacting subjects at follow-up

Patients who had agreed to be in the study were contacted again, 12 months after recruitment. The meeting between the research team and the patients was again arranged through the THC and the VCDA, with data collection at the village clinic or in the home. Multiple visits were made to the villages as necessary to obtain a high response at follow-up.

### Data collected

Information was collected by face-to-face interviews, using a questionnaire developed for this study. This included questions on date of diagnosis, use of health care facilities, medication, diabetic care given in the last 12 months and perceived impact of diabetes. Subjects were also asked about diet, smoking, alcohol use, employment and education. They completed two quality of life scales (the SF-12 and EQ-5D), for which there were established Chinese (Mandarin) versions [9, 10]: these were administered aurally for illiterate subjects. Participants were weighed and their height measured for the calculation of BMI. Capillary (fingertip) blood taken to measure HbA1c (using a DCA vantage analyzer), an indicator of blood glucose over the preceding weeks.

The questionnaire and tests were repeated at the 12 month follow-up interview, omitting items (education, height) unlikely to have changed.

### Outcome measures

1. ***Control of diabetes*** was measured by the value of HbA1c at baseline and follow-up: this measure reflects blood glucose concentration over the previous 3 months, with a higher level indicating worse control of diabetes.
2. ***Quality of diabetes-related care*** was reflected in:
  i). whether the patient had been seen for diabetes at a higher level (county) hospital or only at the local township hospital;
  ii). the number of recommended checks (0–4) reported by the patient. These were examinations, carried out in the last 12 months as part of their health care, of blood glucose (to assess diabetic control), urine (to look for renal damage), eyes (to look for diabetic retinopathy) and feet (to look for skin lesions);
  iii). the total number of hospital visits for diabetes made in the previous 12 months: in Anhui province at this time village clinic doctors could not prescribe diabetic medication so all diabetic care entailed hospital visits.
3. ***Quality of life*** was measured by
  i) the physical and mental component summary scores of the SF-12 [9]. Scores were normalized to a mean of 50 and range 0–100: high scores indicated better health;
  ii) the health score from the visual analogue scale of the EQ-5D on a scale 0–100 with high scores indicating better health [10].
4. ***Economic burden*** was reflected in a self-report that the cost of treatment for diabetes was greatly or very greatly a burden to themselves and their families.

### Statistical methods

A substantial numbers of those registered as having been approved for a CDS card reported at baseline that they had not yet been notified of the decision. Because of this, the main analysis was restricted to matched pairs in which one patient (the case) reported having received their CDS card at baseline and the other (the referent) reported never having applied for a card by the time of the follow-up interview. Within this subset of the population, the design was multi-level with repeat measures on the same subject, with cases matched to referents and both cases and referents clustered within county. In this analysis we used a multi-level logistic model (GLLAMM within STATA) to examine the benefit (or risk) associated with being a member of the chronic disease scheme. We first considered whether those not applying for the scheme had a more adverse change between baseline and follow-up in markers of health and wellbeing than those in the scheme throughout. In a secondary analysis, we considered whether those who joined the scheme between baseline and follow-up were at less at risk (did better) than those who applied but had not (yet) been approved. Again a multi-level logistic model was used, allowing for clustering within county but without taking account of the original matched design.

## Results

A total of 2052 cases recorded as being in the CDS were extracted. Of these, 514 were not approached because the target had already been reached, 434 were not used because no referent could be identified and 182 were not at home when the team visited. A further 253 had moved away to live or work, or were staying with relatives in another village, 36 were in villages where the village doctor refused, 21 had died, nine were the wrong age and three refused: 602 were recruited for the study. Of 1440 referents identified but not used, 851 were not needed because a closer match was found, 272 already had a CDS card, 201 were the wrong age, 93 were not at home when the team visited, 21 denied having diabetes and two refused: 602 patients with diabetes but without a card were recruited.

Of the 602 pairs recruited at baseline all but 78 subjects (36 cases, 42 referents) were successfully interviewed at follow-up, on average 368 days (range: 300–590 days) after the first interview. There was no significant difference between cases and referents in the reason for no contact (*p =* 0.50). Twenty (10 cases, 10 referents) had died, 32 were working away, 11 had moved away, for seven the house was locked and empty, five refused, two were visiting relatives, and one was in hospital. To facilitate comparison of changes between baseline and follow-up, only the 528 pairs in which both case and referent were contacted twice were included in the analyses reported here.

Although all the cases and none of the referents were recorded in NCMS records as having been issued with chronic disease cards, this did not necessarily coincide with the reports of the subjects, with many cases saying that the result of their application was still pending. Moreover, many of the referents had already started the process of applying. The distribution of cases and referents by self-reports of applying for and receiving the card is shown in Table 2. Only nine cases reported never applying for the card although a further 35 said that they had not applied (or did not know if they had applied) at baseline. Similarly only six referents reported at baseline they had received the card although 48 said that they had applied.

**Table 2:** Self-reported timing of application for, and reception of, a chronic disease card (*N* = 1056)

	NCMS reports card				NCMS reports no card			
	Card received				Card received			
Application for card	Never	Before baseline	After baseline	Total	Never	Before baseline	After baseline	Total
Never	9	0	0	9	383	0	0	383
After baseline	3	0	32	35	43	0	54	97
≤12 months before baseline	24	123	71	218	13	1	13	27
>12 months before baseline	9	239	18	266	10	5	6	21
Total	45	362	121	528	449	6	73	528

Initial inspection of the data suggested that the values of the outcome measures at the time of the baseline questionnaire were related to the recency of the CDS application (Table 3). Those who had applied in the 12 months before the baseline interview reported undergoing more types of monitoring tests, more hospital visits and greater proportions visiting a high level hospital in the last 12 months than those either not applying or applying more than 12 months previously: their HbA1c, at 7.69, was lower (better) than that of either those who had not applied (8.03) or those who had applied earlier (8.10) and this difference was unlikely to have arisen by chance (*p =* 0.03). Those not applying for the CDS by baseline had somewhat better quality of life as reflected in the physical and, particularly, the mental health component score of the SF-12, and were very much less likely to report that they were greatly burdened by the cost of treatment.

**Table 3:** Outcome measures at baseline by timing of application

	Timing of CDS application			
Outcome measure	Not by baseline	≤12 months before baseline	>12 months before baseline	*p*=0.03
HbA1c: mean (SD)	8.03 (1.99)	7.69 (1.82)	8.10 (1.87)	
Seen at county or higher level hospital: %	53.6	81.6	65.9	0.00
Number of types of test: mean (SD)	1.28 (0.74)	1.62 (0.81)	1.42 (0.77)	0.00
Number of hospital visits: mean (SD)	2.94 (3.80)	5.48 (5.71)	4.93 (4.34)	0.00
SF-12 physical: mean (SD)	34.4 (8.9)	33.8 (8.1)	33.0 (7.9)	0.07
SF-12 mental: mean (SD)	39.6 (11.1)	37.9 (11.6)	37.7 (10.9)	0.03
EQ-5D health scale: mean (SD)	57.6 (14.4)	58.4 (14.3)	56.4 (14.3)	0.27
Cost of treatment ‘greatly burdensome’ %	38.2	48.6	57.1	0.00
*N*	524	245	287	–

(*N* = 1056)

The subsequent analysis compared only those who had joined the scheme before baseline with those who had never applied. Using a matched analysis as originally planned, it first considers 258 matched pairs (76 male and 182 female) in which both NCMS records and self-report confirmed that the case had received the card by baseline and in which the referent reported that they had not applied for a chronic disease card for diabetes by the time of the 12 month follow-up. This sub-population allows us to estimate the ongoing impact of application and membership through changes in outcome measures between baseline and follow-up.

The baseline characteristics of these 258 pairs (Table 4) demonstrate that they had been well matched on age. There were differences between cases and referents in the time since diagnosis, with both men and (particularly) women in the scheme reporting that their diagnosis had been at a more distant date than those who had not applied for the scheme. Cases were also much more likely to be using insulin at baseline (65 cases but only 9 referents): the large majority of both cases and referents not using insulin reported using Western oral diabetic treatments with only 6.6 % of cases and 20.5 % of referents using no medication or only traditional Chinese treatments. Few other differences were seen. Both male and female cases had a somewhat higher mean body mass index (BMI) than referents and male referents were more likely to report that they drank alcohol. The final columns of Table 4 show the odds ratios and 95 % confidence intervals (CI) from a multilevel logistic model allowing for the initial matching. It confirms that the use of insulin is the baseline factor most clearly differentiating those in the scheme, with small additional differences, particularly for women, in years since diagnosis and BMI.

**Table 4:** Baseline characteristics of 258 matched pairs (in scheme or not applied throughout)

Baseline characteristics	Case	Referent	p=	OR^a^	95 % CI^a^
Age					
men: mean (SD)	58.1 (7.9)	54.4 (8.8)	0.87	0.98	0.93–1.03
women: mean (SD)	59.1 (7.2)	59.4 (6.6)	0.62	0.99	0.95–1.03
Years since diagnosis^b^					
men: mean (SD)	6.2 (5.8)	4.8 (5.8)	0.16	1.02	0.95–1.09
women: mean (SD)	6.9 (5.2)	4.5 (3.9)	0.00	1.10	1.04–1.17
Body mass index					
men: mean (SD)	26.0 (4.4)	24.8 (3.2)	0.06	1.00	0.99–1.01
women: mean (SD)	25.8 (4.0)	25.2 (3.6)	0.10	1.06	1.00–1.13
Use of insulin					
Men: %	22.4	6.6	0.00	4.43	1.30–15.13
Women: %	26.4	2.2	0.00	15.16	4.45–51.67
Smoker now					
Men: %	42.1	42.1	1.00	1.04	0.51–2.12
Women: %	0.5	2.2	0.72	0.60	0.05–4.30
Alcohol now					
Men: %	26.3	40.8	0.08	0.61	0.28–1.31
Women: %	2.2	4.9	0.26	0.30	0.07–1.33
Working for money					
Men: %	42.1	43.4	1.00	1.32	0.57–3.02
Women: %	7.7	12.6	0.25	0.53	0.23–1.23
Family income ‘lower than most’					
Men: %	56.6	47.4	0.33	1.60	0.76–3.35
Women: %	55.5	61.0	0.34	0.64	0.39–1.04
Very low literacy					
Men: %	22.4	18.4	0.69	1.35	0.55–3.27
Women: %	67.0	73.6	0.21	0.88	0.51–1.52
*N*					
Men	76	76	–	–	–
women	182	182	–	–	–

^a^Adjusted for all other factors in table 3 in a multi-level logistic model

^b^Among cases 3 men and 15 women could not recall the year of diagnosis

Among referents 2 men and 13 women could not recall the years of diagnosis

The difference between cases and referents on the eight outcome measures, at baseline and follow-up are shown for this study group in Table 5: probabilities of the univariate comparison between cases and referents are given, followed by odds ratios adjusted for use of insulin, years since diagnosis and BMI as recorded at baseline. No difference was seen in the extent to which blood glucose control had been achieved, as reflected in HbA1c, at baseline or follow-up. However, the markers of quality of diabetes care were found to favour those in the scheme: they were more likely to report having been seen at a higher level hospital (county level or higher) for their diabetes in the previous 12 months, both at baseline and follow-up; they also reported at baseline and follow-up that more of the 4 types of recommended checks had been carried out and that they made more hospital visits for their diabetes. In contrast the quality of life measures were better for those not in the scheme, particularly at baseline. The referents had higher ratings, at both time points, on both the physical and mental component scales of the SF-12 and had a better perception of their overall health, as reflected in their visual analogue responses on the EQ-5D, although this difference was much less in evidence when the responses were adjusted for use of insulin and time since diagnosis. Finally those in the scheme felt more greatly burdened by treatment costs at both contacts. None of the measures on which change could readily be measured (HbA1c, tests, hospital visits, quality of life scales) suggested a magnitude of change that differed between cases and referents: both groups reported fewer tests and better quality of life at follow-up and fewer in each group reported feeling greatly burdened by the cost of treatment.

**Table 5:** Outcome measures for 258 matched pairs (in scheme or not applied throughout)

Outcome measures	Case	Referent	p=	OR^a^	95 % CI^a^
HbA1c					
Baseline: mean (SD)	8.06 (1.90)	7.97 (1.90)	0.60	0.96	0.89–1.06
Follow up: mean (SD)	8.07 (1.97)	8.10 (1.90)	0.83	0.92	0.83–1.02
Change: mean (SD)	0.00 (1.50)	0.13 (1.28)	0.30	0.93	0.81–1.07
Seen at county or higher level hospital					
Baseline %	74.8	50.4	0.00	2.83	1.87–4.29
Follow up %	67.1	41.9	0.00	2.58	1.74–3.82
Number of test types					
Baseline: mean (SD)	1.46 (0.73)	1.20 (0.67)	0.00	1.67	1.25–2.23
Follow up: mean (SD)	1.25 (0.66)	1.02 (0.64)	0.00	1.56	1.14–2.14
Change: mean (SD)	−0.21 (0.89)	−0.17 (0.83)	0.65	0.91	0.73–1.15
Number of hospital visits					
Baseline: mean (SD)	5.47 (5.64)	2.50 (3.58)	0.00	1.24	1.15–1.33
Follow up: mean (SD)	5.82 (5.68)	2.58 (3.92)	0.00	1.15	1.09–1.21
Change: mean (SD)	0.35 (6.44)	0.09 (4.65)	0.59	1.00	0.97–1.04
SF–12 Physical					
Baseline: mean (SD)	32.4 (7.7)	35.2 (9.1)	0.00	0.97	0.95–1.00
Follow up: mean (SD)	35.1 (7.2)	36.5 (7.5)	0.03	0.98	0.96–1.01
Change: mean (SD)	2.7 (8.9)	1.3 (9.6)	0.08	1.02	0.99–1. 04
SF–12 Mental					
Baseline: mean (SD)	37.5 (10.5)	40.7 (11.3)	0.00	0.98	0.96–1.00
Follow up: mean (SD)	39.4 (10.8)	41.5 (10.9)	0.03	0.99	0.97–1.00
Change: mean (SD)	1.9 (12.5)	0.8 (12.5)	0.33	1.00	0.99–1.02
EQ–5D Health scale					
Baseline: mean (SD)	56.2 (13.9)	59.5 (13.5)	0.01	0.99	0.98–1.04
Follow up: mean (SD)	62.2 (8.0)	64.5 (9.5)	0.00	0.98	0.96–1.00
Change: mean (SD)	2.7 (8.9)	1.3 (9.6)	0.08	1.00	0.99–1.02
Cost of treatment burdensome					
Baseline: %	57.8	33.7	0.00	2.06	1.39–3.05
Follow up: %	33.7	16.3	0.00	1.95	1.22–3.10
**N**	258	258	–	–	–

^a^Adjusted for insulin, years since diagnosis and BMI at baseline in a multilevel logistic model allowing for clustering within pairs

This analysis gave information on how membership of the scheme impacted on outcome measures, but not (since all the cases were in the CDS at baseline) on changes associated with newly receiving the card. The subgroup most able to provide information on this comprised the 35 cases and 97 referents who said at baseline they had not applied for the card, but that they had applied by the follow-up (Table 2). Of these 85 received the card before the 12 month follow-up questionnaire and 46 did not. It appears (Table 6) that those who did not get the card showed a small deterioration in diabetes control (HbA1c) between baseline and follow-up whereas those who received the card had achieved better control by the second assessment. No other difference was seen between the groups except on the physical component score of the SF-12, where those receiving the card showed greater improvement.

**Table 6:** Outcome measures for those who applied after baseline: card received or not by follow up

Outcome measures	Received	Not received	p=	OR^a^	95 % CI^a^
HbA1c					
Baseline: mean (SD)	8.38 (2.38)	7.92 (1.53)	0.24	1.09	0.90–1.31
Follow up: mean (SD)	7.95 (1.91)	8.12 (1.86)	0.73	0.96	0.79–1.17
Change: mean (SD)	−0.43 (1.51)	0.20 (1.44)	0.02	0.74	0.56–0.99
Seen at county or higher level hospital					
Baseline %	65.1	71.7	0.56	0.78	0.35–1.76
Follow up %	64.0	67.4	0.85	0.77	0.34–1.72
Number of test types					
Baseline: mean (SD)	1.47 (0.89)	1.41 (0.78)	0.74	0.96	0.58–1.59
Follow up: mean (SD)	1.24 (0.61)	1.26 (0.49)	0.87	0.89	0.46–1.72
Change: mean (SD	−0.22 (0.96)	−0.15 (0.89)	0.69	0.98	0.63–1.53
Number of hospital visits					
Baseline: mean (SD)	4.42 (4.67)	3.57 (4.08)	0.30	1.03	0.94–1.13
Follow up: mean (SD)	5.44 (5.71)	4.11 (5.08)	0.19	1.04	0.97–1.13
Change: mean (SD	1.02 (6.91)	0.54 (5.80	0.70	1.01	0.95–1.07
SF–12 Physical					
Baseline: mean (SD)	31.51 (8.27)	34.53 (8.79)	0.05	0.96	0.92–1.00
Follow up: mean (SD)	34.95 (7.66)	33.92 (7.60)	0.46	1.02	0.97–1.07
Change: mean (SD)	3.44 (7.74)	−0.61 (9.90)	0.01	1.06	1.01–1.10
SF–12 Mental					
Baseline: mean (SD)	37.93 (10.13)	36.59 (11.68)	0.49	1.01	0.98–1.05
Follow up: mean (SD)	38.51 (10.24)	35.43 (10.47)	0.11	1.03	0.99–1.07
Change: mean (SD)	0.57 (10.99)	−1.16 (11.42)	0.40	1.01	0.98–1.05
EQ–5D Health scale					
Baseline: mean (SD)	54.93 (14.74)	53.13 (14.95)	0.51	1.01	0.99–1.04
Follow up: mean (SD)	59.40 (10.24)	62.30 (9.65)	0.12	0.97	0.93–1.01
Change: mean (SD)	4.47 (12.88)	9.17 (14.82)	0.06	0.97	0.94–1.00
Cost of treatment greatly burdensome					
Baseline: %	54.7	45.7	0.36	1.37	0.64–2.92
Follow up: %	33.7	34.8	0.90	0.83	0.37–1.83
N	86	46	–	–	–

^a^Adjusted for insulin use at baseline in a multilevel logistic model allowing for clustering within county

## Discussion

This study was designed to evaluate effects of the Anhui Province chronic disease scheme on the health and wellbeing of those in the province. We used diabetes as an exemplar condition as there is a biological marker (HbA1c) that acts an indicator of the level of control achieved by a patient with diabetes. As it was not feasible to randomly allocate patients with diabetes to enroll in the scheme, the comparison was primarily between those who were enrolled in the scheme throughout and those who did not apply, with a secondary, prospective, comparison of change between baseline and follow-up of those who applied since baseline. The results did not suggest that those who had been enrolled in the scheme throughout the study had better control of their diabetes than those who never applied for the scheme. However was some evidence that better control of diabetes was present in those who had recently applied for the scheme and, particularly those who had recently been accepted, although it is not known whether this improvement would be sustained.

There are a number of ways in which interpretation of these data are difficult. First, there is some uncertainty about the date at which a patient was formally enrolled in the scheme, with many registered by the NCMS office as having been approved for a card reporting that they had not yet received it. As such, the self-reported date of application is used here as a proxy, but we are left in some uncertainty about the extent to which investigations and advice associated with the (self-funded) application process influenced the outcome measures of diabetic control, diabetic care, quality of life and economic burden: it is difficult to disassociate the potential impact of application from any health or other benefit arising from use of the chronic disease card and the eligibility for reimbursements once it is issued.

Second, as in all observational studies in which the intervention (here membership of the CDS) is not randomly assigned, there is a risk of selection bias: although the province had a process in place to identify all patients with diabetes and to offer enrolment in the CDS to all who qualified, this was not fully achieved at the time of this study. Most (>80 %) of decisions to apply for the scheme were made at the suggestion of the treating physician and it seems possible that those who came forward as an early candidate for the scheme may have had diabetes that was more difficult to control: among the 256 pairs included in the primary analysis, those in the scheme (particularly among the women) had been diagnosed at an earlier date. They were somewhat more likely to report heart disease (12.8 % against 8.9 %) and renal disease (3.9 % against 0.8 %) at baseline that those not in the scheme. They were also more likely to be using insulin when recruited for the study (greater than 20 % of those in the scheme reported insulin use at baseline) but it is not known if this was a cause or result of CDS membership. While such measured differences between groups can be allowed for in the statistical analysis (as, for example, in the odds ratios included in Table 5) there may also be unmeasured differences that cannot be included as confounders (a problem also referred to as endogeneity in this context [11]). Matching patients in the scheme with referents who were not, but were of the same age, sex and village may have helped to reduce the effects of such unmeasured confounding, but is unlikely to have eliminated it entirely.

The high proportion of those in the CDS who felt that the cost of diabetic treatment was greatly burdensome is consistent with earlier studies showing that out of pocket (OOP) expenses increased along with the greater use of services associated with the introduction of the NCMS or other types of health insurance. [5] Rural households including members with chronic disease such as diabetes have been shown to have much greater OOP outpatient expenses than household with no chronic disease, and to be at increased risk of catastrophic health expenditures, even after reimbursements [12]. Although in the present study partial reimbursement for outpatient care was available to those in the scheme, it is of interest that among the 258 who had been in the scheme since baseline, only 176 (68 %) had received any reimbursements in the previous 12 months. The most common reason cited for not being reimbursed was that the drugs they were prescribed were not on the essential drugs list. In many, if not most, cases the treating physician will have known that the patient was in the CDS.

Finally, it is unclear why those studied reported feeling less burdened and a higher quality of life at the follow-up interview. This may have been from greater familiarity with the research team, a more realistic assessment of the role of the research (which may initially have been seen as facilitating access to health benefits) or unrelated changes in health care provision or other facilities for rural inhabitants: without a referent group, these improvements might have erroneously been attributed to the CDS itself.

The question of whether health insurance improves health, rather than simply access to health services, is not readily answered but the finding of better (but still not ideal) control of blood glucose amongst those recently approved for the scheme does suggest that reception of (and early compliance with) medical treatment within the scheme may improve diabetic control, at least in the short term: this finding, together with the absence of effect of CDS membership for those longer in the scheme, implies a need for continued active follow-up of those recently accepted for the scheme, to reinforce and maintain this early success. Systematic review [13] and meta-analysis [14] suggest that changes in the team or management plans for patients with type 2 diabetes can be effective in increasing control of blood glucose and in decreasing long term risk of cardiovascular disease [15]. Longer follow-up of this cohort of patients with diabetes would be needed to determine whether CDS membership was associated with fewer life-threatening complications of the disease.

## Conclusion

On-going membership of the CDS was associated with increased use of services but this did not appear to result in better management of blood glucose or improved quality of life. Those who had recently joined the scheme had signs of improvement, suggesting a need for active follow-up to maintain and reinforce early gains.

## Abbreviations

BMI: Body mass index
CDS: Chronic disease scheme
NCMS: New cooperative medical scheme
THC: Township health centre
VCDA: Village clinic doctor administrator
